# Supplementary material for: Exploring the Aβ Plaque Microenvironment in Alzheimer’s Disease Model Mice by Multimodal Lipid-Protein-Histology Imaging on a Benchtop Mass Spectrometer
Source: Pharmaceuticals (Basel). 2025 Feb 13;18(2):252. doi: 10.3390/ph18020252 (PMC11860057; doi:10.3390/ph18020252)
Supplement: Supplementary file 1 [file pharmaceuticals-18-00252-s001.zip › pharmaceuticals-3368112-supplementary.pdf]

Supplementary Figures

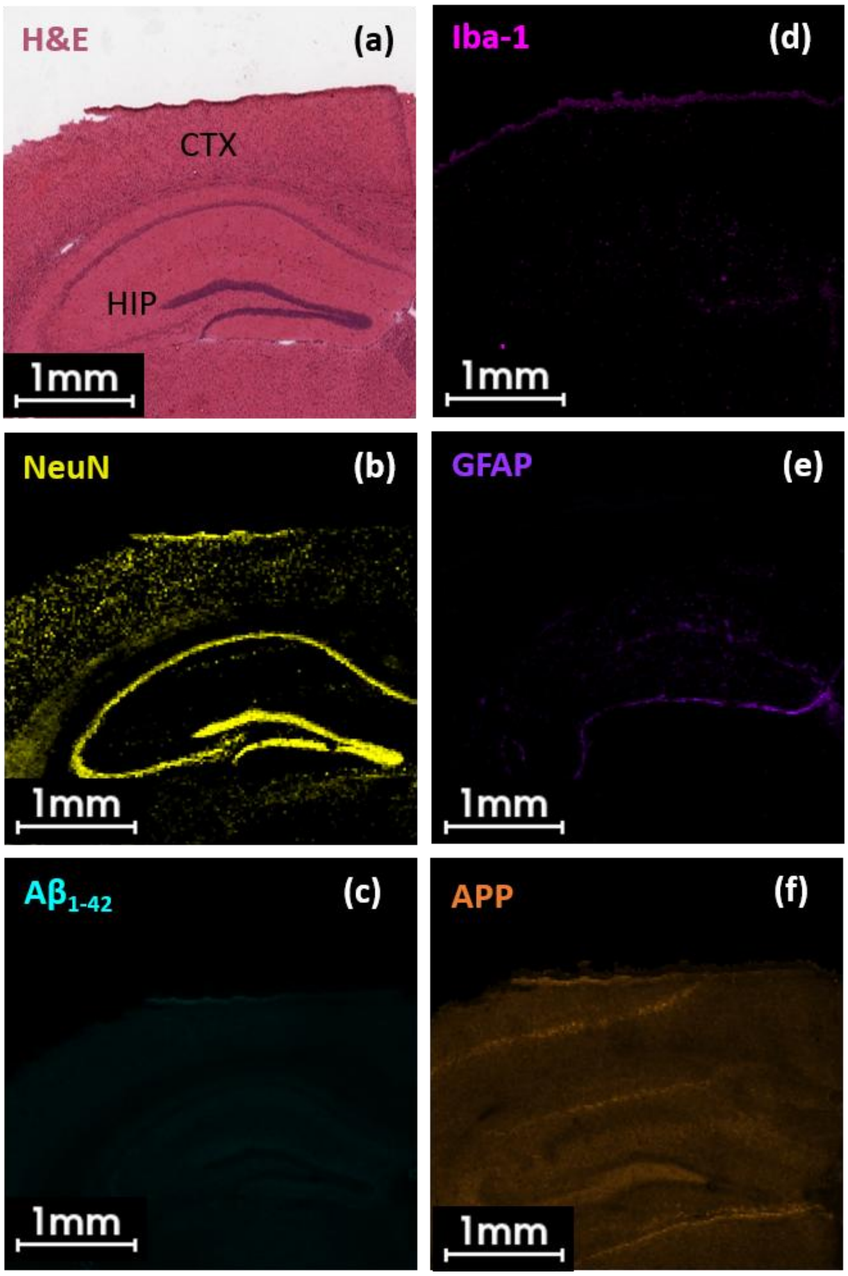

Supplementary Figure S1. Endogenous protein biomarker distribution in WT mouse brain visualized by MALDI-HiPLEX IHC

(a) H&E staining highlights distinct brain structures, *e.g.*, the hippocampus and cortex (CTX). HiPLEX-MALDI IHC images of five PCMTs visualizing marker distribution in a coronal WT mouse brain section. Single ion images of brain structural marker **(b)** NeuN (Neuronal nuclei, *m/z* 1309.0 $[M+H]^+$), (**c)** AD amyloid-β_{1-42} peptide ($A\beta_{1-42}$, *m/z* 1771.5 $[M+H]^+$), (**d)** microglia marker Iba-1 (*m/z* 960.1 $[M+H]^+$),**(e)** glial fibrillary acidic protein (GFAP, *m/z* 1011.9 $[M+H]^+$) and (**f)** amyloid precursor protein (APP, *m/z* 1723.6 $[M+H]^+$).

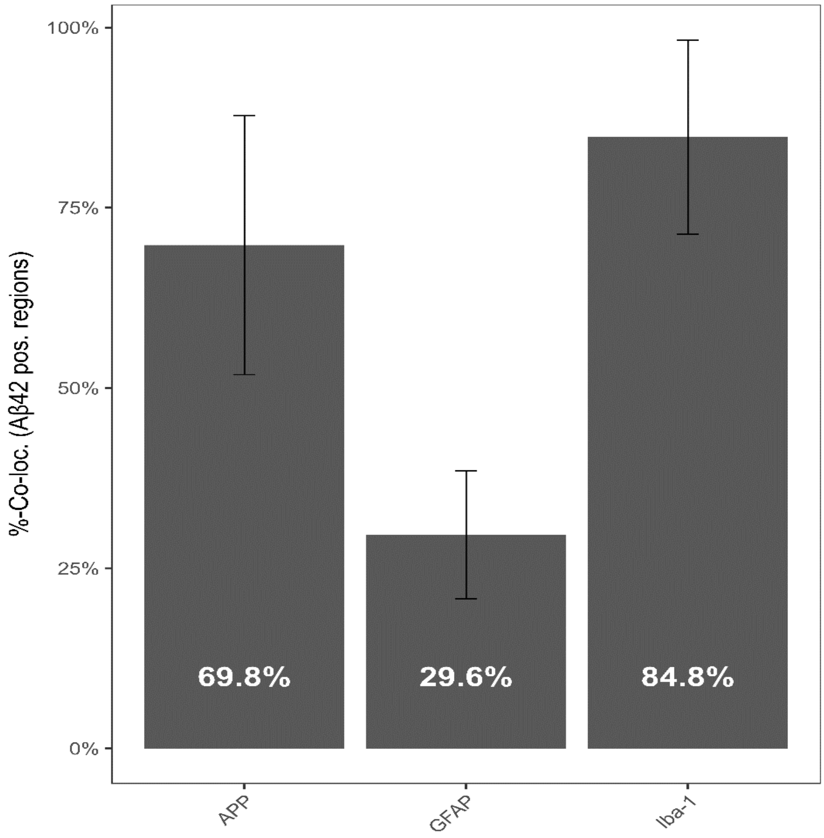

Supplementary Figure S2. Evaluation of co-localization of important AD biomarkers in the APPPS1 mouse brain

Degree of co-localization between the amyloid plaque marker $A\beta_{1-42}$ and the other three immune markers (APP: 69.8 ± 18%, GFAP: 29.6 ± 9%, Iba-1: 84.8 ±13%; n=3 tissue slices). The co-localization is determined on a pixel-by-pixel basis, and positivity is assessed through the utilization of the t-point threshold algorithm, as implemented within the PlaquePicker R-package (Enzlein et al., 2020).

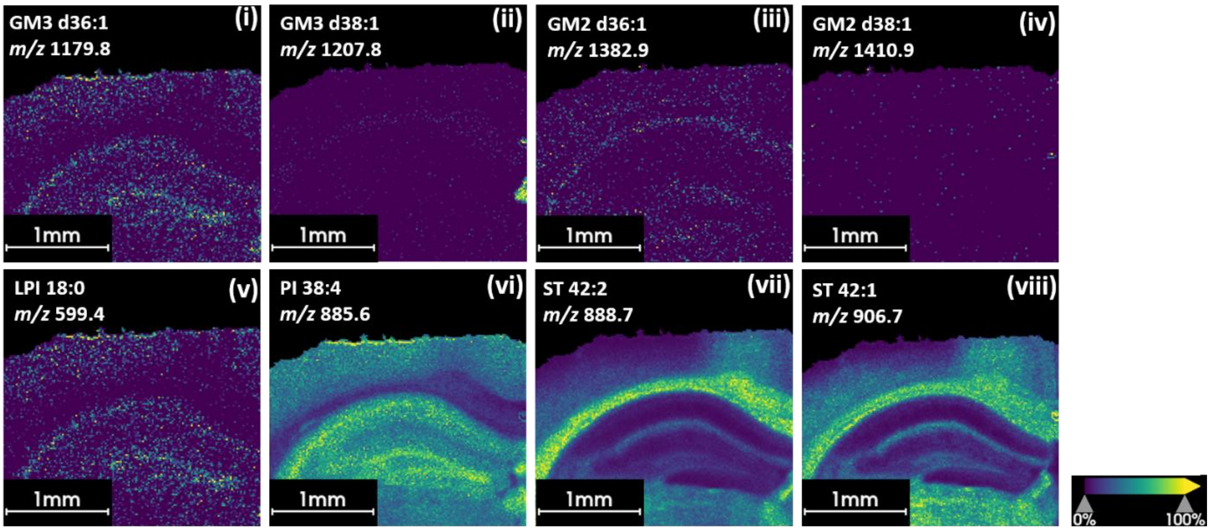

Supplementary Figure S3. MALDI lipid imaging reveals distribution of selected lipids in WT mouse brain tissue

MALDI lipid ion images of eight different lipid features in negative ionization mode detected in WT mouse brain **(i-viii)**. **(i)** Ganglioside GM3 d36:1 (*m/z* 1179.8, $[M-H]^-$), **(ii)** GM3 d38:1 (*m/z* 1207.8, $[M-H]^-$), **(iii)** GM2 d36:1 (*m/z* 1382.9, $[M-H]^-$), **(iv)** GM2 d38:1 (*m/z* 1410.9, $[M-H]^-$), **(v)** lysophosphatidyl-inositol 18:1 (LPI, *m/z* 599.4, $[M-H]^-$), **(vi)** phosphatidylinositol 38:4 (PI *m/z* 885.6, $[M-H]^-$), **(vii)** sulfatide 42:2 (ST d42:2;O2, *m/z* 888.7, $[M-H]^-$) and **(viii)** sulfatide 42:1 (ST d41:2;O2, *m/z* 906.7, $[M-H]^-$).

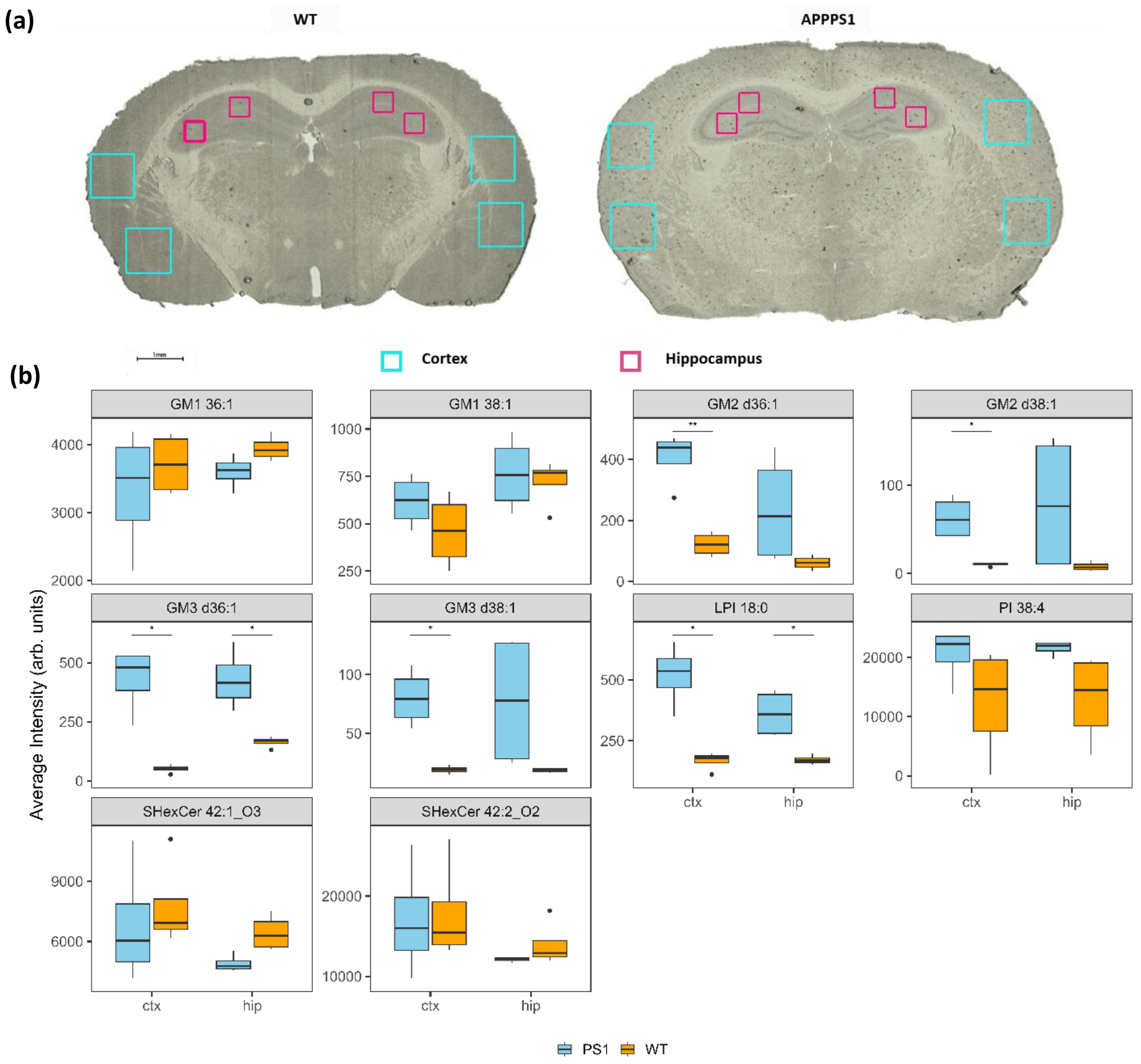

Supplementary Figure S4. Statistical analysis of lipid candidate accumulation in the APPPS1 and WT mouse brain.

(a), Relative abundance of lipid candidates are compared between APPPS1 (pink) and WT (blue) mouse brain tissue by comparing mean values from 4 different regions of interest per tissue type and region. **(b),** The relative differences of the lipid abundancy are compared in the cortex (ctx) as well as the hippocampus (hip).
Unpaired t-test with Benjamini-Hochberg correction, n = 4, * p-value < 0.05, ** p-value < 0.01.

(i)

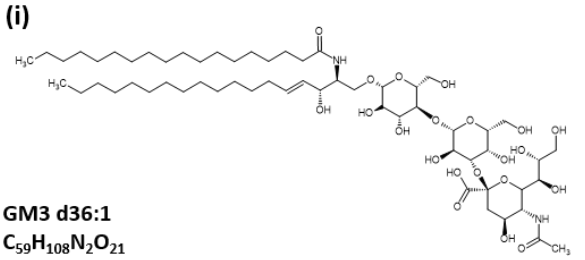

GM3 d36:1
$C_{59}H_{108}N_2O_{21}$
1180.74 [M]

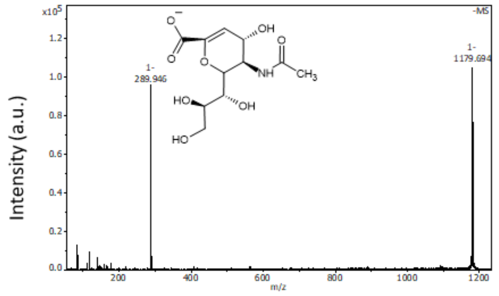

(ii)

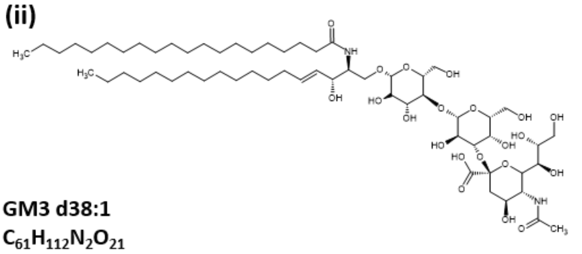

GM3 d38:1
$C_{61}H_{112}N_2O_{21}$
1208.77 [M]

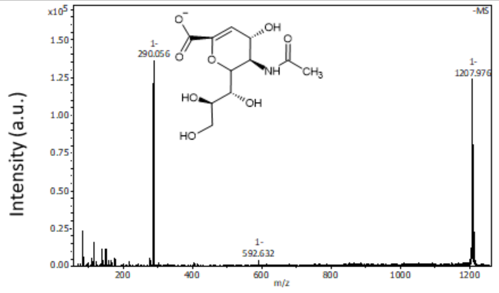

(iii)

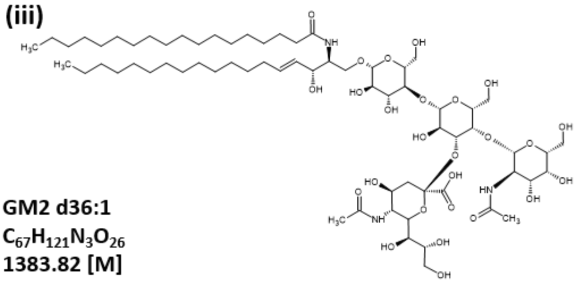

GM2 d36:1
$C_{67}H_{121}N_3O_{26}$
1383.82 [M]

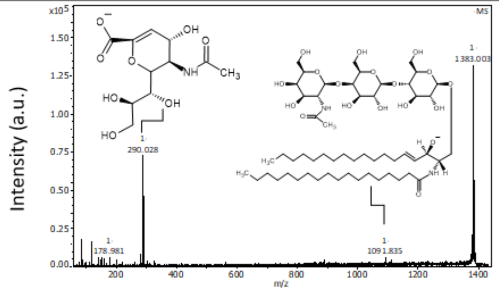

(iv)

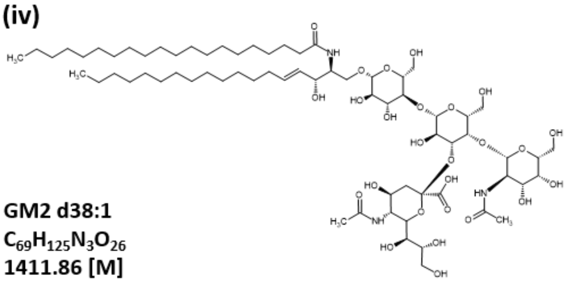

GM2 d38:1
$C_{69}H_{125}N_3O_{26}$
1411.86 [M]

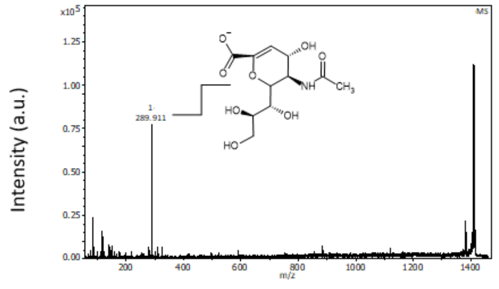

(v)

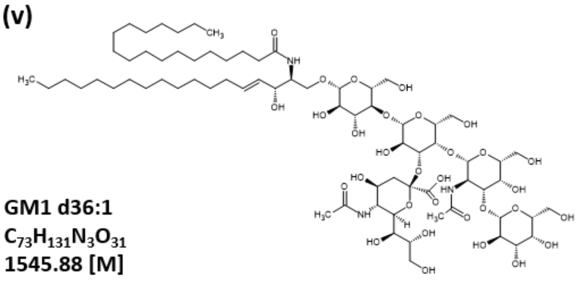

GM1 d36:1
$C_{73}H_{131}N_3O_{31}$
1545.88 [M]

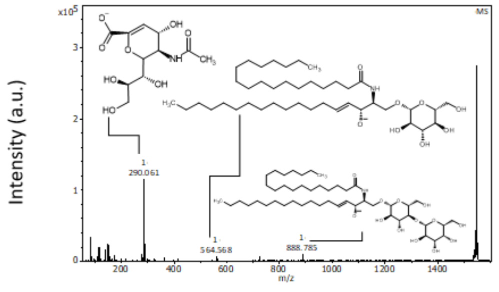

(vi)

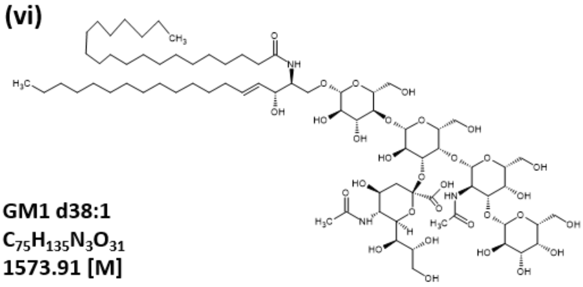

GM1 d38:1
$C_{75}H_{135}N_3O_{31}$
1573.91 [M]

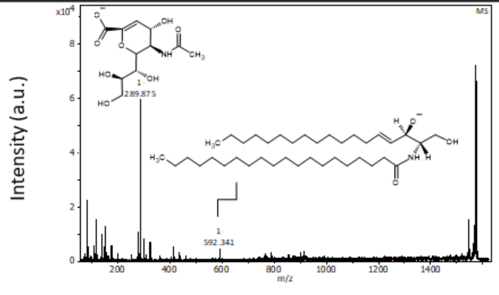

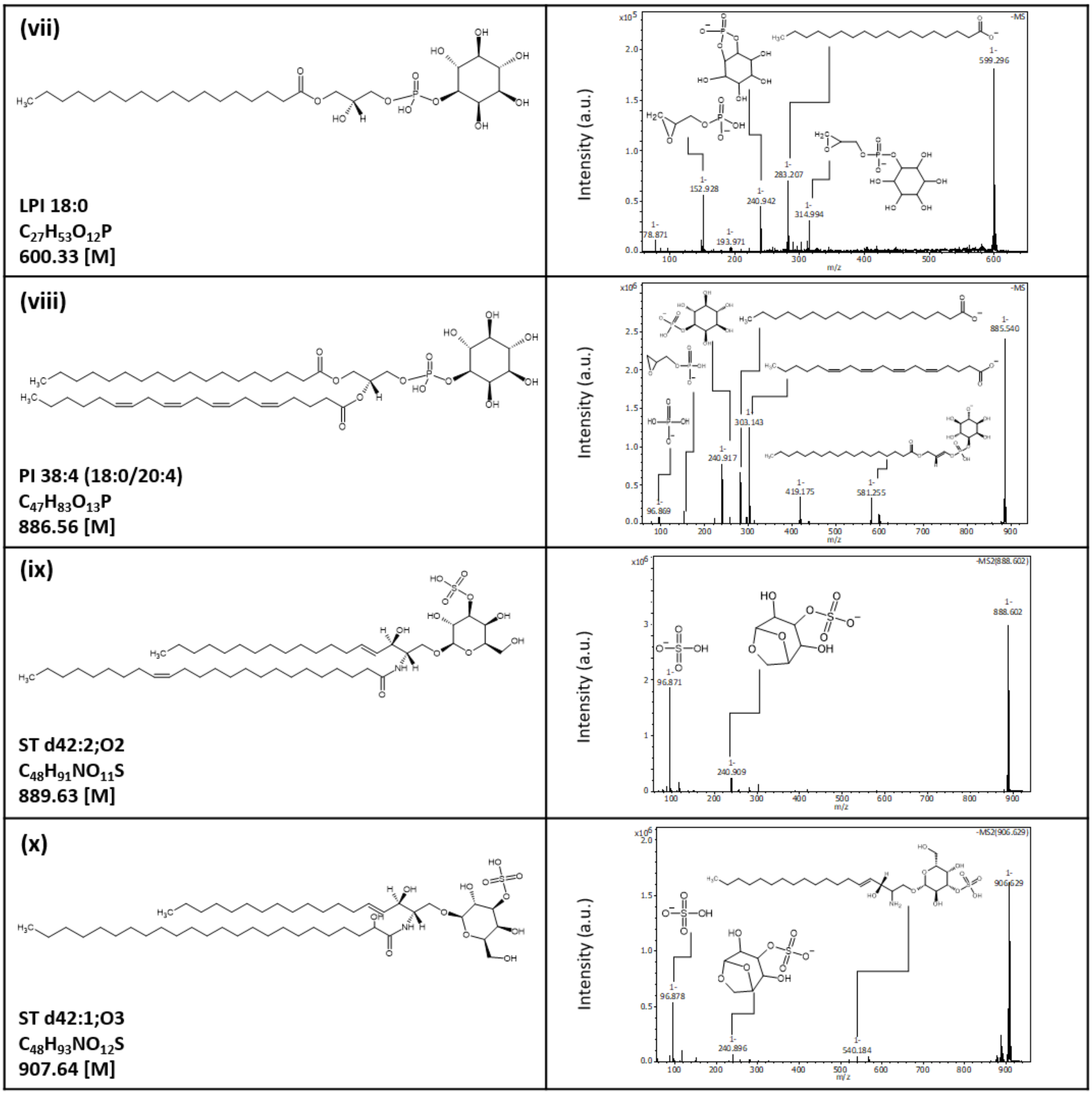

Supplementary Figure S5. Identification of candidate AD lipid biomarkers by TOF/TOF on-tissue fragmentation analysis using a bench top mass spectrometer

Summary of the chemical structure of the putative lipid precursor of interest (left) and the respective MS^2 fragmentation spectra from single shot data acquisition with identified lipid fragments (right) obtained on a neofleX MALDI-TOF/TOF benchtop instrument **(i-x)**.
